# Intermuscular Hibernoma of the Thigh: A Diagnostic and Surgical Challenge Managed With Preoperative Embolization

**DOI:** 10.7759/cureus.100602

**Published:** 2026-01-02

**Authors:** Shirin Moosakutty, Afrah Fathima Karimbanakkal Edakkattu

**Affiliations:** 1 Radiology, IQRAA International Hospital and Research Centre, Kozhikode, IND; 2 Medicine, Government Medical College Manjeri, Manjeri, IND; 3 Medicine, Kerala University of Health Sciences, Thrissur, IND

**Keywords:** brown fat, hibernoma, intermuscular neoplasm, liposarcoma, soft tissue tumor

## Abstract

This report describes a rare case of a large intermuscular hibernoma of the thigh associated with an intralesional arteriovenous malformation (AVM), presented to highlight the diagnostic challenges and the role of preoperative embolization in facilitating safe surgical excision. A 50-year-old female presented with a painless, progressively enlarging swelling over the anterior aspect of the left thigh for one year. On examination, a firm, non-tender, slightly mobile mass measuring approximately 20 × 15 cm was noted in the intermuscular plane, with intact neurovascular function. MRI revealed a well-defined fatty lesion with multiple tortuous vascular flow voids, hyperintense on T1 and heterogeneously hyperintense on T2/STIR, with heterogeneous post-contrast enhancement. CT angiography demonstrated multiple feeding vessels arising from the lateral branch of the profunda femoris artery and an intra-lesional AVM. Selective embolization of the feeding vessels was performed preoperatively to reduce intraoperative blood loss. Surgical excision under spinal anesthesia revealed a well-encapsulated, highly vascular mass situated between the sartorius and rectus femoris/vastus muscles, which was removed en bloc with minimal bleeding. Histopathology showed multivacuolated adipocytes with granular cytoplasm and central nuclei, without atypia, confirming a benign hibernoma. The postoperative period was uneventful, and no recurrence was observed at follow-up.

## Introduction

Soft tissue tumors of adipocytic origin are among the most frequently encountered mesenchymal neoplasms in clinical practice. While lipomas and liposarcomas dominate the differential spectrum, hibernomas, benign tumors derived from brown adipose tissue (BAT), remain exceedingly rare, accounting for less than 2% of all benign adipocytic tumors [1].

The thigh represents one of the more frequent peripheral sites for hibernoma development, yet large intermuscular variants are exceptionally uncommon. The tumor’s intrinsic hypervascularity often complicates intraoperative management due to the potential for significant bleeding, thereby underscoring the importance of accurate preoperative imaging and vascular mapping [2]. Advanced imaging modalities, including magnetic resonance imaging (MRI) and computed tomography angiography (CTA), have improved diagnostic precision and assist in preoperative planning by demonstrating characteristic features such as well-defined fatty masses with internal septations and prominent vascular channels [3]. However, despite these advances, precise diagnosis often remains elusive until definitive histopathological examination, as hibernomas are frequently misinterpreted as angiolipomas or well-differentiated liposarcomas.

This report describes the case of a 50-year-old female presenting with a large intermuscular hibernoma of the left thigh, managed successfully through preoperative embolization followed by complete surgical excision.

## Case presentation

Patient information and clinical history

The patient presented in March 2024 to the Department of Radiology at Aster Malabar Institute of Medical Sciences (MIMS) Hospital, Kozhikode, Kerala, India. This 50-year-old female presented with a painless, progressively enlarging swelling over the anterior aspect of the left thigh for approximately one year. There was no history of trauma, infection, or systemic illness. She reported mild discomfort and cosmetic concerns due to the increasing size of the lesion but no pain, restricted mobility, or neurological symptoms. Her past medical and surgical history was unremarkable, and there were no known allergies or family history of similar tumors (see Table 1).

**Table 1:** Timeline of events

Stage	Event
Initial symptom onset	Patient first noticed swelling over the anterior aspect of the left thigh.
Clinical presentation (after ~339 days)	Patient presented with a large thigh swelling (~20 × 15 cm). MRI demonstrated a fatty lesion with internal vascular channels; CT angiography revealed multiple feeding vessels with an intralesional arteriovenous malformation.
Preoperative intervention	Pre-operative embolization of the feeding branches of the profunda femoris artery.
Surgical management	Surgical excision of intermuscular thigh mass under spinal anesthesia; ~15 × 10 cm (958 gram), highly vascular, completely excised.
Postoperative course	Post-operative recovery uneventful with analgesics, anti-inflammatory drugs, and a compression bandage.
Discharge and follow-up	Discharged stable with medications (Hifenac P, Chymoral Forte, Topgrip bandage). Advised OPD follow-up.

Clinical findings

On local examination, a well-defined swelling measuring approximately 20 × 15 cm was observed on the anteromedial thigh. It was firm, non-tender, and slightly mobile, with no local rise in temperature or skin changes. The swelling was located in the intermuscular plane, with preserved overlying skin and no vascular bruit. Neurovascular examination revealed intact distal pulses and normal motor-sensory function. No regional lymphadenopathy was noted, and systemic examination findings were unremarkable.

Diagnostic assessment

Imaging Studies

MRI of the left thigh revealed a well-circumscribed intermuscular mass showing hyperintensity on T1 and heterogeneous hyperintensity on T2/short tau inversion recovery (STIR), with multiple internal flow voids suggestive of vascular channels. Post-contrast enhancement was heterogeneous.

CT angiography demonstrated multiple feeding vessels with a dominant arterial supply from the lateral branch of the profunda femoris artery and an intra-lesional arteriovenous malformation (AVM). The differential diagnosis included angiolipoma and hibernoma.

Laboratory Investigations

Routine hematology revealed mild microcytic hypochromic anemia (Hb: 10.6 g/dL) and mild eosinophilia (7%, absolute eosinophil count (AEC): 0.37 × 10⁹/L), with all other parameters within normal limits (see Table 2).

**Table 2:** Hematology investigations performed MCV: Mean corpuscular volume; MCH: Mean corpuscular hemoglobin; MCHC: Mean corpuscular hemoglobin concentration; RDW-CV: Red cell distribution width-coefficient of variation; MPV: Mean platelet volume; PDW: Platelet distribution width

Test Name	Result	Normal Range	Interpretation
Hemoglobin (Hb)	10.6 g/dL	12.0-16.0 g/dL	Low – anemia
Packed Cell Volume (PCV)	33.8%	36-46%	Low
RBC Count	4.42 × 10¹²/L	3.8-4.8 × 10¹²/L	Normal
MCV	76.5 fL	80-96 fL	Low – microcytosis
MCH	23.9 pg	27-32 pg	Low – hypochromia
MCHC	31.3 g/dl	32-35 g/dL	Slightly low
RDW-CV	13.5 %	11.6-14.0%	Normal
Platelet Count	245 K/uL	150-450 K/uL	Normal
PDW	16.0 fl	8.5-14.1 fL	High
MPV	9.0 fl	7.4-10.3 fL	Normal
Total Leukocyte Count (TLC)	5.8 K/uL	4.0-11.0 K/uL	Normal
Neutrophils (%)	53%	40-70%	Normal
Lymphocytes (%)	33%	20-40%	Upper normal
Eosinophils (%)	7%	1-6%	Mild eosinophilia
Monocytes (%)	7%	2-8%	Normal
Basophils (%)	0%	0-1%	Normal
Absolute Neutrophil Count	3.1 × 10⁹/L	2.0-7.0 × 10⁹/L	Normal
Absolute Lymphocyte Count	1.9 × 10⁹/L	1.0-3.0 × 10⁹/L	Normal
Absolute Eosinophil Count	0.37 × 10⁹/L	0.02-0.5 × 10⁹/L	Slightly high
Absolute Monocyte Count	0.4 × 10⁹/L	0.2-0.8 × 10⁹/L	Normal

Therapeutic intervention

Preoperative Embolization

Given the lesion’s marked vascularity, selective embolization of the feeding branches from the profunda femoris artery was performed under fluoroscopic guidance. This effectively reduced the tumor’s vascularity and minimized intraoperative bleeding risk.

Surgical Procedure

**Figure 1:**
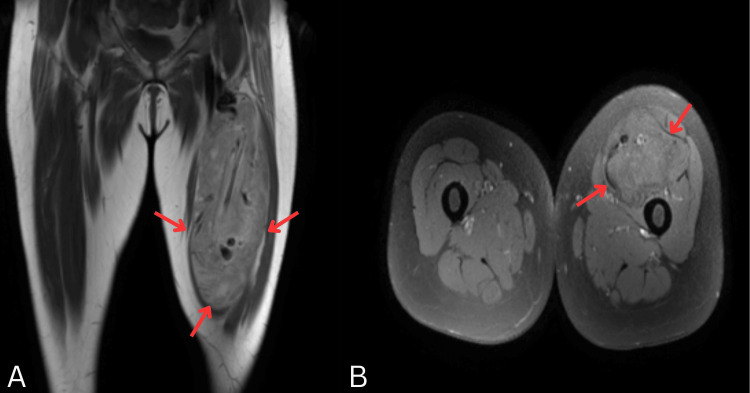
MRI of the left thigh demonstrating an intermuscular mass (A) Coronal T1-weighted image shows a well-defined heterogeneous intermuscular lesion in the anteromedial compartment (arrows), containing areas of fat signal and internal vascular channels. (B) Axial T1-weighted image highlights a large heterogeneous mass with prominent vascular structures in the left thigh (arrows), in contrast to the normal appearance of the right thigh.

**Figure 2:**
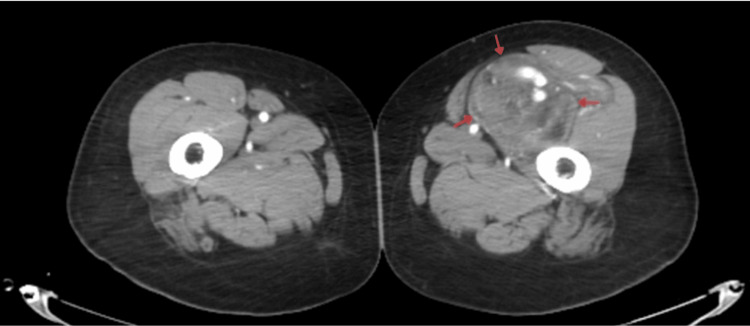
CT angiogram of the thighs (axial view) A large, enhancing intermuscular mass is present in the anteromedial compartment of the left thigh. Multiple dilated feeding vessels and prominent intralesional vascular channels are seen, arising from branches of the profunda femoris artery.

**Figure 3:**
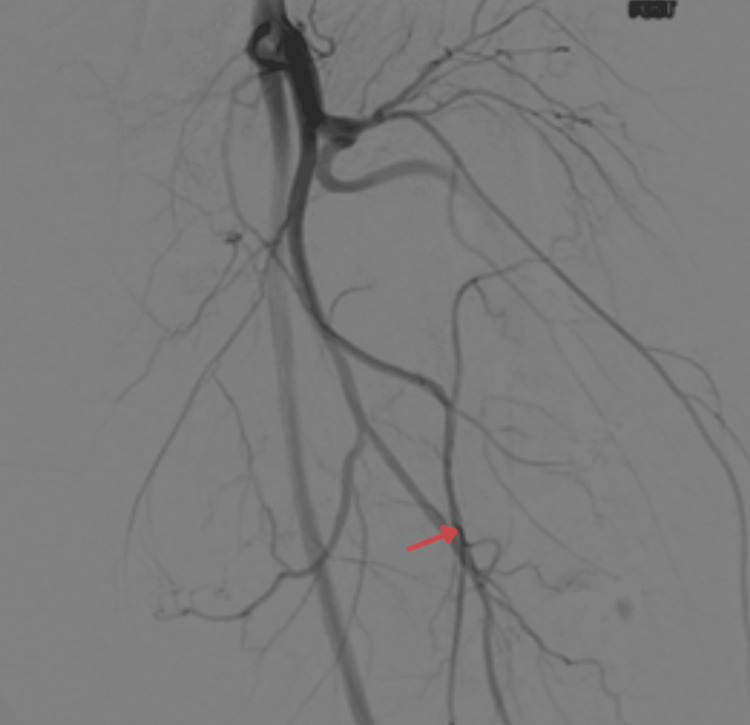
Pre-operative digital subtraction angiography (DSA) of the left thigh The study demonstrates multiple hypertrophied feeding vessels supplying the intermuscular mass in the anteromedial compartment. Selective embolization of branches arising from the profunda femoris artery was performed prior to surgery to reduce anticipated intraoperative blood loss.

**Figure 4:**
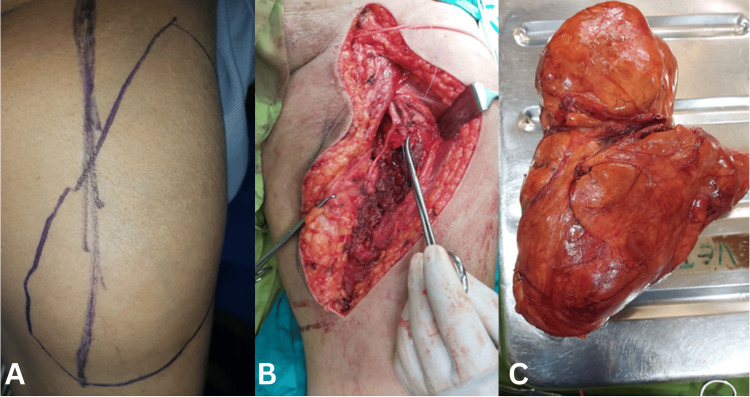
Surgical planning, intraoperative findings, and excised specimen *Scales and scoring were free to use (A) Preoperative marking over the anteromedial thigh outlining the planned incision and the vascular course to guide safe dissection. (B) Intraoperative exposure showing a large intermuscular mass with multiple feeding vessels identified and controlled prior to excision. (C) Gross specimen demonstrating a well-circumscribed, encapsulated, lobulated fatty tumor measuring approximately 17.5 × 8.6 × 7.1 cm*.

Definitive surgical excision was performed under spinal anesthesia. A well-encapsulated intermuscular mass measuring 15 × 10 cm was identified between the sartorius (medially) and rectus femoris/vastus muscles (laterally). Multiple feeding vessels were ligated and controlled, allowing complete en bloc excision with minimal blood loss. The surrounding muscle planes and neurovascular structures were preserved (see Figures 1-4).

Postoperative care

The postoperative period was uneventful. The wound was closed in layers and supported with a Topgrip compression bandage. The patient received Tab Hifenac P (Aceclofenac + Paracetamol) - twice daily for three days, as needed for pain relief, Tab Chymoral Forte - thrice daily for five days to reduce inflammation and edema. The patient was discharged on postoperative day three in stable condition.

Pathological findings

**Figure 5:**
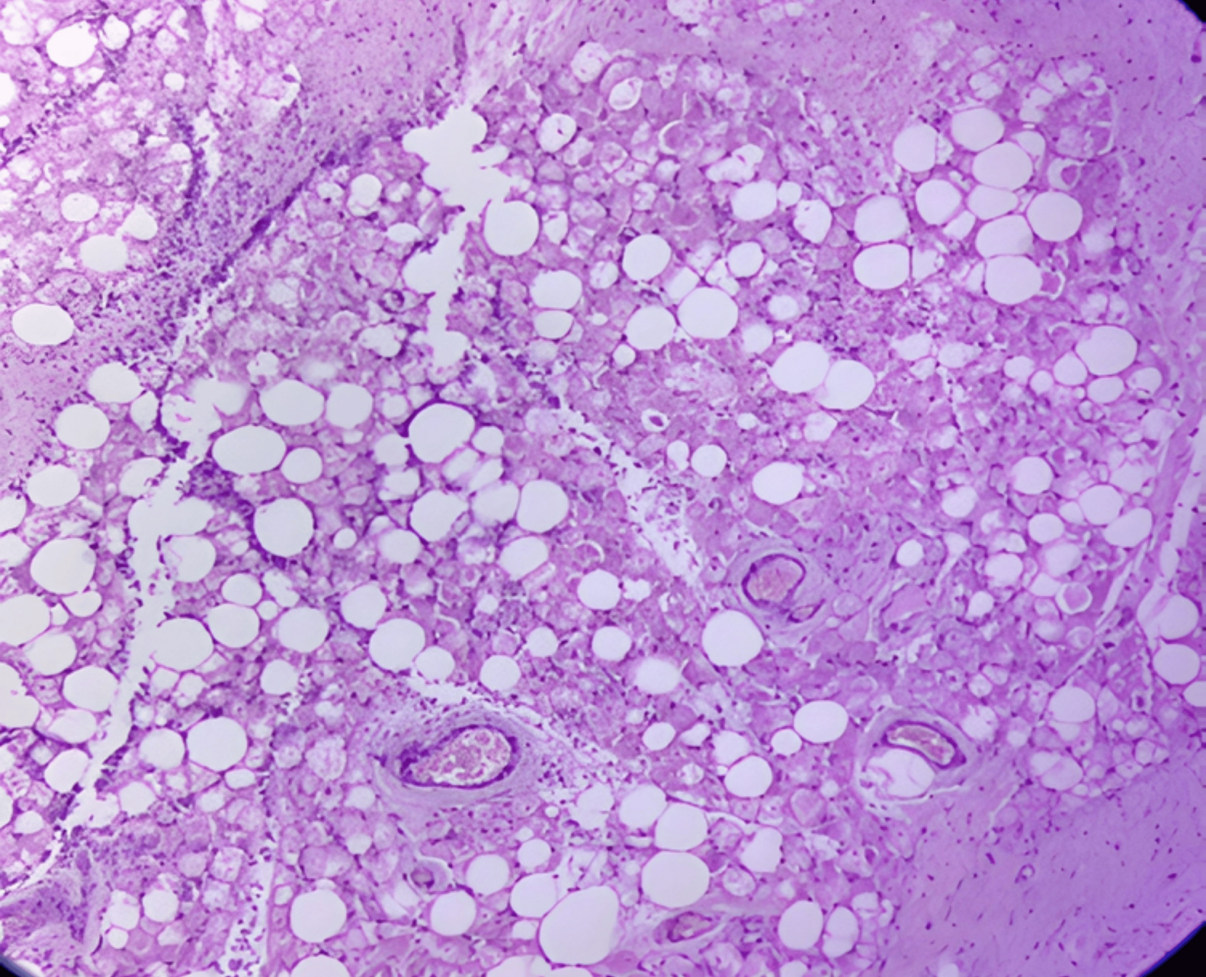
Histopathology of the excised left thigh mass (H&E, ×40) Microscopic section shows the sheets of large polygonal cells with abundant multivacuolated granular cytoplasm and small central nuclei, admixed with univacuolated adipocytes. No cytological atypia, mitosis, or necrosis is seen.

Gross examination showed a lobulated yellow mass measuring 17.5 × 8.6 × 7.1 cm, with focal hemorrhagic areas. Microscopic evaluation (H&E, ×40) revealed polygonal, multivacuolated adipocytes with granular eosinophilic cytoplasm and central nuclei, admixed with univacuolated adipocytes. No atypia, mitosis, or necrosis was observed. The final diagnosis is benign hibernoma (negative for malignancy) (see Figure 5).

Outcome and follow-up

The postoperative course was smooth, with complete recovery and no complications such as hematoma or infection. The patient reported significant relief in limb heaviness and cosmetic improvement. She was advised to have regular follow-up at three- and six-month intervals. As of the last review, there was no evidence of recurrence.

## Discussion

Hibernomas are rare benign tumors derived from brown adipose tissue and account for less than 2% of all benign lipomatous neoplasms. They are typically slow-growing, well-circumscribed, and hypervascular lesions that often mimic malignant lipomatous tumors both radiologically and metabolically. The present case of an intermuscular hibernoma of the thigh with an AVM and preoperative embolization adds valuable insight into the diagnostic and surgical spectrum of this uncommon entity.

The thigh represents the most frequently reported site for hibernomas, comprising approximately 30% of all documented cases [4]. In the current case, the lesion originated within the anterior thigh intermuscular compartment. We searched for the most cited recent similar case reports. Faenza et al. described a large (21 × 13 × 11 cm) hibernoma in a young male involving the medial thigh and compressing the obturator nerve, and Gauci et al. reported an intramuscular forearm lesion in a 60-year-old male [4,5]. Whalen et al., in contrast, documented a smaller subscapularis hibernoma that presented incidentally as a fluorodeoxyglucose (FDG)-avid lesion mimicking metastasis [6].

Imaging plays a pivotal role in differentiating hibernomas from other lipomatous and vascular lesions. MRI typically reveals a well-defined mass with high T1 and T2 signal intensities and incomplete fat suppression, reflecting the admixture of brown and white fat [6]. Flow voids or vascular channels are a frequent finding due to the tumor’s intrinsic vascularity, as observed in both the current case and those described by Faenza et al. [4]. However, our case demonstrated an additional angiographic feature, an intra-lesional AVM, which has seldom been reported. This prompted preoperative CT angiography and selective embolization of feeding vessels from the profunda femoris artery, minimizing intraoperative bleeding. Such interventional management is rarely documented in previous literature, underscoring its significance in surgical planning for highly vascular hibernomas [4].

Histopathologically, all reviewed cases, including the present one, exhibited classic features of hibernoma multivacuolated adipocytes with granular eosinophilic cytoplasm, absence of atypia, and a fine capillary network. Faenza et al. [4] classified their case as the lipoma-like variant, while Gauci et al. [5] and Whalen et al. [6] described the typical subtype, aligning with our findings. Immunohistochemical or molecular studies, such as MDM2 negativity by fluorescence in situ hybridization (FISH), as performed by Faenza et al. [4], may be valuable when differentiating hibernoma from well-differentiated liposarcoma. In our case, the combination of MRI and CT angiographic features with characteristic histopathology was sufficient for confirmation [4-6].

Surgical excision remains the gold standard for management. Complete resection provides a definitive cure, and recurrence is exceedingly rare even with large lesions [4-6]. The postoperative outcomes across all four cases, including the present report, were uniformly favorable, with no local recurrence or functional deficit. The additional step of preoperative embolization in our patient represents an important refinement for managing vascular hibernomas, facilitating a bloodless field and safe excision.

In summary, the reviewed literature demonstrates that hibernomas can present at various anatomical sites with distinct radiological mimics-vascular in our case, neural in Faenza et al. [4], and metabolic in Whalen et al. [6]. The present case is distinguished by its size, intermuscular location, and the use of preoperative embolization to address pronounced vascularity. Awareness of these features is critical to avoid misdiagnosis as liposarcoma or vascular malformation, to ensure optimal imaging-based planning, and to achieve excellent surgical outcomes.

Learning points

Hibernoma, though benign, can mimic liposarcoma or vascular malformations on imaging due to its fatty composition and marked vascularity. Multimodal imaging, particularly MRI and CT angiography, is essential for accurate diagnosis and surgical planning in vascular soft-tissue tumors. Preoperative embolization is a valuable and under-reported adjunct that significantly reduces intraoperative blood loss in large, vascular hibernomas.

## Conclusions

This case demonstrates that intermuscular hibernoma of the thigh, though benign, can pose a diagnostic and surgical challenge due to its vascularity and resemblance to malignant lipomatous tumors. Preoperative embolization, followed by complete surgical excision, ensures a safe and curative outcome. Awareness of this rare entity is essential to prevent misdiagnosis and overtreatment.
